# Limitation of the Lytic Effect of Bacteriophages on *Salmonella* and Other Enteric Bacterial Pathogens and Approaches to Overcome

**DOI:** 10.1155/ijm/5936070

**Published:** 2025-05-15

**Authors:** Chuan-Wei Tung, Dita Julianingsih, Anna Phan, Christa Canagarajah, Zabdiel Alvarado-Martínez, Debabrata Biswas

**Affiliations:** ^1^Department of Animal and Avian Sciences, University of Maryland, College Park, Maryland, USA; ^2^Biological Sciences Program-Molecular and Cellular Biology, University of Maryland, College Park, Maryland, USA

**Keywords:** antibiotic-resistance, bacteriophage, endolysin, food safety, phage therapy, *Salmonella enterica*

## Abstract

Bacteriophages (phages) have emerged as promising agents for combating bacterial pathogens, including nontyphoidal *Salmonella enterica* (*S. enterica*), the most common foodborne pathogen worldwide. The emergence of antimicrobial-resistant (AMR) *S. enterica* poses a severe healthcare issue. Nowadays, many countries worldwide have banned antibiotics for animal feeds or additives, and various strategies have been developed and gained popularity for their potential to address *S. enterica* infection. Among these strategies, phage therapy shows more promise because of its ability to specifically target bacterial pathogens without disrupting the beneficial microbiota or animal/human cells. Phages are viruses that rupture host cells through the lysis of phage-encoded endolysin proteins. Nonetheless, phages also face various challenges, including phage resistance, gene transduction, serovar diversity, and the immune response of animal/human organisms, which limit the efficacy of *S. enterica*. Due to this limitation of phages, endolysin, as a lytic protein for bacterial cells derived from phages, has been demonstrated as another promising solution against various bacterial pathogens, including AMR. This review is aimed at discussing the benefits and limitations of phage therapies and exploring the promising potential of phage-encoded endolysins in controlling *S. enterica*.

## 1. Introduction

The global population surge and growing demand for food, especially animal products, have significantly increased foodborne disease outbreaks [1]. These illnesses, linked to over 200 diseases caused by consuming food contaminated with bacteria, viruses, parasites, or chemicals, pose a major global health concern. According to the estimate, 600 million people suffer from foodborne infections, leading to approximately 420,000 deaths worldwide [2]. Most foodborne diseases are associated with diarrheal illnesses due to consuming foods contaminated with pathogens. Diarrheal diseases rank as the eighth leading cause of death among all age groups and the fifth leading cause among children under 5 years old globally [3, 4]. Bacterial pathogens such as *Salmonella enterica* (*S. enterica*), *Campylobacter jejuni*, *Listeria monocytogenes*, and *Escherichia coli* have been recognized as major foodborne bacterial pathogens worldwide [2] and in the United States [1]. Among these bacterial pathogens, nontyphoidal *S. enterica* is the second most common foodborne bacterial pathogen in the United States and other developed countries, and the second common in the European Union/European Economic Area (EU/EEA) [5, 6]. It causes approximately 1.9 million cases in the United States and 78 million cases globally annually [2, 5, 6].

### 1.1. *Salmonella* as an Enteric Pathogen

*S. enterica* is a Gram-negative, rod-shaped, facultative anaerobe that belongs to the Enterobacteriaceae family. More than 2600 serovars or serotypes of this bacterium have been identified [7]. Based on the major serotypes of *S. enterica*, it can be divided into typhoidal *S*. *enterica* and nontyphoidal *S*. *enterica* [8]. Typhoidal *S. enterica* includes *S.* serotype Typhi (*S.* Typhi) and *S. enterica* serotype Paratyphi (*S.* Paratyphi), which cause typhoid fever and paratyphoid fever in humans, respectively [9, 10]. In contrast, common nontyphoidal *S*. *enterica* strains include various serovars such as *S. enterica* Typhimurium (*S.* Typhimurium), *S. enterica* serovar Enteritidis (*S.* Enteritidis), *S. enterica* serovar Newport (*S*. Newport), and *S. enterica* serovar Heidelberg (*S*. Heidelberg). These serovars are associated with foodborne gastroenteritis, leading to symptoms like fever, diarrhea, vomiting, abdominal pain, nausea, and bacteremia. While their impact is generally nonfatal, infections can be lethal for young children, the elderly, immunocompromised individuals, or those with chronic conditions [4, 11].

Most nontyphoidal *S*. *enterica* serovars colonize many other warm-blooded animals, particularly chickens [12]. As a result, outbreaks involving these pathogens are commonly linked to poultry or poultry products. The predominant serotypes involved in salmonellosis outbreaks vary from year to year. According to a recent report, *S.* Enteritidis, *S.* Typhimurium, *S.* Heidelberg, and *S.* 1,4,[5],12:i:- are commonly linked to foodborne salmonellosis outbreaks in the United States [13] and *S.* Enteritidis, *S.* Typhimurium, *S.* 1,4,[5],12:i:-, and *S.* Infantis in the EU/EEA [5]. A recent study conducted in the Maryland-DC region of the United States revealed that the prevalence of *S*. *enterica* was 7.80% at the preharvest level and 1.91% in organically grown postharvested food products [14].

Transmission of nontyphoidal *S*. *enterica*, such as *S.* Enteritidis, *S.* Heidelberg, and *S.* Typhimurium occurs through several routes, including the consumption of contaminated foods or drinks, close contact with contaminated environments or animals, and horizontal or vertical transmission among animals [12]. In particular, partially or uncooked eggs, chickens, chicken/egg dishes, and chicken/egg products are significant contributors to *S. enterica* infection [15]. *S. Enteritidis* also can penetrate the eggshell and lead to the contamination of egg contents through infection of the reproductive organs [16]. Additionally, *S. Enteritidis* can spread among chickens, pigs, and other animals through contaminated feed or free-range environments [12].

Furthermore, the increasing trend of antimicrobial resistance (AMR) bacterial pathogens poses a significant concern [14, 17, 18]. Over the past few decades, the overuse and misuse of antimicrobials in both humans and animals have resulted in AMR in enteric pathogens, particularly *S. enterica* [19]. AMR bacteria can be transferred from animals to humans through the consumption of animal products or contact with animals containing AMR pathogens [19]. In 2019, it was estimated that approximately 1.27 million deaths occurred globally each year due to AMR bacteria [20]. AMR strains of *S. enterica* now pose a substantial threat to both animal and human health worldwide, contributing to an annual economic burden of $35 billion in the United States [21]. It is estimated that AMR strains of nontyphoidal *S. enterica* are responsible for approximately 212,500 infections and 70 deaths annually in the United States [22].

Many countries, including the United States and the EU, have prohibited the use of antibiotics as feed additives in animal agriculture to address the AMR issue [21, 23, 24]. To satisfy the rising demand from consumers for products free of antibiotics, this restriction made it easier to expand the production of organic and free-range poultry [25]. However, these alternative farming methods are now more susceptible to foodborne microbial contamination, particularly AMR bacteria, because of the decreased management of outside surroundings in free-range poultry production [26]. In addition, some pathogens for chickens, such as *Salmonella pullorum* and *Salmonella gallinarum*, are commonly present in preharvest and postharvest [27]. Investigating alternate tactics like bacteriophage therapy or bacteriophage-encoding enzymes becomes more and more critical in these changing difficulties.

### 1.2. Potential Strategies to Control *S. enterica*

In addition to the prohibition of using antibiotics as feed additives in many countries, many conventional poultry productions have shifted toward alternative antimicrobials to prevent colonization of zoonotic bacterial pathogens in livestock including poultry [28]. Among these alternative antimicrobials, such as prebiotics, probiotics, synbiotics, plant-derived antimicrobial compounds, essential oils, vaccination, and bacteriophage therapies, are gaining popularity for controlling colonization of *S. enterica* in poultry [11, 28]. Probiotics are a group of nonpathogenic microorganisms, such as *Lactobacillus* and *Bifidobacterium*, that provide health benefits to their host when administered in sufficient quantities [11, 29, 30]. Probiotics must meet safety and efficacy for their host, possess immunomodulatory properties, effectively colonize the intestinal epithelium, withstand bile salts and low pH, and maintain both phenotypic and genetic stability [29]. Prebiotics are nondigestible components that undergo selective fermentation, leading to beneficial changes in the composition and activity of the gastrointestinal microbiota [31]. Prebiotics, such as mannan-oligosaccharides (MOSs) and fructo-oligosaccharides (FOSs), have been shown to enhance beneficial gut bacteria and prevent *S.* Enteritidis infection by altering gut microbial diversity in poultry [32, 33]. Synbiotics, which combine probiotics and prebiotics, further enhance the survival of beneficial bacteria in the gastrointestinal tract, improving immune responses and overall health in poultry [34]. However, the effectiveness of synbiotics depends on factors like the specific strains used and the conditions of the gastrointestinal environment [35].

Plant-derived natural antimicrobial compounds, also known as phytobiotics, such as garlic, oregano, and cinnamon, have been shown to improve nutrient absorption, feed intake, and immune function in poultry [36]. These plant-derived metabolites, specifically flavonoids/phenolics have, demonstrated antimicrobial activity against *Salmonella* [37, 38]. Polyphenolic components, such as berry pomace extracts (BPEs), a byproduct with shell and seeds after juicing of blueberry or blackberry fruits, are shown to significantly inhibit the growth of *S.* Typhimurium and its virulent functions [39–41]. Other polyphenolic components, such as gallic acid, protocatechuic acid, and vanillic acid, are identified as properties that inhibit the growth of *S.* Typhimurium in vegetables and are potential candidates as alternative antimicrobial agents [42–44]. Essential oils (volatile oils) are aromatic compounds with distinct flavors and fragrances. They are extracted from various parts of plants and contain various compounds such as alcohols, acetones, phenolic acids, terpenes, aldehydes, and esters, all of which can act as critical antimicrobial agents or nutritional supplements [31]. One common type of essential oil, citrus oil, effectively prevents the contamination/colonization of *Salmonella* in poultry and poultry products [45].

Vaccinations are widely used to boost immune responses and prevent *Salmonella* shedding [46]. However, each vaccination has its limits regarding safety and effectiveness. The most common challenges are improving animal vaccine administration and costs for animal industries [47]. Each vaccination has its own set of limits in terms of safety and effectiveness. Bacteriophage (phage) therapy is gaining significant attention as a promising approach to combat bacterial infections. This review is aimed at assessing the progress of phage therapy and the potential of phage-encoded endolysins in controlling *S. enterica* infection. These advancements present new opportunities to overcome the limitations of conventional treatments and enhance food safety.  Table 1 lists the benefits and challenges of these alternative strategies for controlling *Salmonella*.

## 2. Bacteriophages and Their Effectiveness

### 2.1. Bacteriophage

Bacteriophages, or phages, are viruses that specifically target and infect bacterial cells rather than animal cells. They were first discovered in 1915 by William Twort, and 2 years later, in 1917, Felix d'Herelle identified their potential to combat bacteria, establishing them as a promising therapeutic agent for treating and preventing bacterial infections in both humans and animals [48]. Due to the development of synthetic antibiotics during World War II, phages lost prominence in the pharmaceutical industry. However, with the emerging AMR bacterial pathogens, phage research has become a promising alternative to replace antibiotics [49].

Phages are the most abundant microbes on Earth, estimated at 10^31^ phage particles, and they can be found ubiquitously in various natural environments where bacteria exist [50]. Phages are highly specific to their hosts/bacteria, typically infecting only a single bacterial species or specific strains within a species, regardless of their AMR pattern. Phages display remarkable diversity in size, appearance, and genetic organization [51]. Their size ranges from 24 to 200 nm, containing either single or double-stranded DNA or RNA [52]. Notably, there are certain phages referred to as “giant phages” or “jumbo phages” due to their exceptionally large genome sizes, ranging from 200 to 500 kbp [53]. These giant phages include *Salmonella* phage SPN3US, Proteus Phage 7, Enterobacteria phage SEGD1, and *Salmonella* bacteriophage-1252 [54]. Many of the gene functions within these jumbo phages remain unknown, and their essential roles require further investigation [52]. Therefore, agricultural animal producers, specifically poultry farmers, are searching for bacteriophage as an alternative strategy to prevent zoonotic pathogens in livestock gut and control contamination of animal food products/environment to limit foodborne bacterial illness [28].

### 2.2. Mechanisms of Phage Infection

Lytic phages, such as the Coliphage T4, initiate infection with the attachment of the phage tails to receptor proteins located at the surface of the host/bacterial cell. This interaction occurs between the tip on the phage tail fiber and various receptor proteins on the bacterial surface, such as outer membrane proteins, peptidoglycan, lipopolysaccharide, teichoic acids, oligosaccharides, flagellum, capsule, Type IV fimbriae, and sex pili, and this interaction allows the phage to inject its genome into the bacterial cell [55]. In general, most narrow host-range phages interact with specific receptor-binding proteins on the surface of a particular bacterial species, allowing them to infect only the target bacterial species [56].

Phages replicate within their host cell and then proceed through two main lifecycles: the lytic and the lysogenic (temperate) lifecycles [57]. During a lytic cycle, a phage genome utilizes the host metabolism and ribosomes for phage DNA replication and protein synthesis. Only lysogenic phages can be converted to the lytic cycle, whereas lytic phages can replicate only through the lytic cycle. At the end of the lytic process, most phages produce two major proteins: holins and endolysins. In addition, other phages may produce proteins such as pinholins, signal-anchor-release (SAR) endolysins, antiholins, and spanins [58], depending on the phage type. These proteins facilitate host cell lysis and the release of progeny phages.

In the lysogenic or temperate cycle, some phages undergo a latent infection, integrating their DNA into the chromosome of the host as a prophage. Instead of lysing the bacterium, the prophage postpones virion production and undergoes replication and multiplication during the bacterial cell replication and binary fission processes. Subsequently, the phage genome is transmitted to daughter bacterial cells without causing their death [59]. During the lysogenic cycle, prophages encode membrane-anchored proteins through “superinfection exclusion (Sie) systems” in the host cell, which block the entry of phage genomes from the same strain or specific phages [60, 61]. Well-characterized Sie systems, such as those in coliphage T4, utilize the Immunity protein (Imm) [62] and Spackle protein (Sp) [63]. Imm alters the phage injection site, while Sp inhibits T4 lysozyme activity, preventing host cell wall degradation and subsequent phage DNA entry [60, 61].

The lysogenic state can be maintained for long periods and converted to a lytic lifecycle, causing lysis of the host cell. This switch is often triggered by exposure to environmental stressors [64], such as the bacterial Son of Sevenless response [65]. It is believed to be a survival tactic employed by phages to escape from host cells that are in danger of dying [66] or to maintain their populations during harsh conditions [67]. However, lysogenic phages may lose their capability over time to be induced or to exit from the bacterial chromosome [68]. Holins are small transmembrane proteins that accumulate in the host cell membrane unit and serve as a lysis “clock” on the length of the lytic cycle. Their accumulation leads to an oligomeric complex that disrupts the bacterial cell inner membrane [69]. At a specific moment, holin triggers the host cell membrane to suddenly become permeable toward endolysin, and it also plays a significant role in the length of the lytic cycle [70]. Another similar functional protein is pinholins, which form small pores (approximately 2 nm in diameter) and lead to membrane depolarization, triggering SAR endolysin activation and degradation of the host cell wall [58].

Endolysins are enzymes phages produce and accumulate in the cytosol during the lysis cycle [71]. Endolysins can be categorized into canonical endolysins and SAR endolysins. SAR endolysins are preanchored to the periplasmic side of the cell membrane. They are released and degrade the cell wall due to membrane depolarization induced by pinholins [58, 72]. In Gram-negative bacteria, spanins are essential for lysing bacteria in inner membrane and outer membrane after the cell wall has been degraded by endolysin or SAR endolysin [58]. Spanins traverse both the inner membrane and outer membrane with two forms: a single molecule as an inner membrane-spanin and a heterodimeric structure as an outer membrane-spanin [73]. Lysis is triggered when spanin complexes are released and accumulate on the inner side of the peptidoglycan layer, followed by fusion of the inner membrane and outer membrane, causing disruption of the cell membranes [74]. Disruption of the cell membranes and cell wall will release newly formed phages from the host cell and start another round of infection in neighboring host cells [59].

Certain phage-derived enzymes, such as integrase and transposase, lead to genetic mutations and bacterial evolution by integrating phage DNA into host chromosomes [64]. Integrases enable site-specific recombination between phage and bacterial attachment sites (attP and attB), making them useful tools for genetic manipulation, particularly in organisms with large genomes like mammals and plants [75]. Transposase is a key contributor to the field of mobile DNA from phage Mu and promotes both replication and integration during the lytic cycle [76]. Although these enzymes provide potential for genetic engineering, their use in medical applications carries associated risks of AMR gene transfer.

### 2.3. Phage Therapy

Phage therapies have been applied successfully in the current application, specifically lytic phages, which can lyse targeted foodborne bacterial pathogens in foods or in livestock gut while leaving the beneficial microflora unaffected. This alternative strategy not only efficiently inhibits pathogenic bacteria and extends the shelf life of foods but also offers advantages such as the absence of harmful antimicrobial residues [77]. “Listex P100,” the first commercial phage product for *L. monocytogenes*, was approved by the FDA in 2006 and is used in ready-to-eat foods and poultry products in the United States [78, 79]. This FDA approval recognized the benefit of phages in producing safer food and established them as generally recognized as safe (GRAS) material [80]. As a result, numerous phage preparations for food applications have since been granted approval in the United States [79–81].  Table 2 presents FDA-approved phage products to control foodborne pathogens from 2006 to 2024. These products have a wide range of applications in meat, poultry, fruits, vegetables, dairy products, and seafood. Of the 17 products, eight specifically target *Salmonella*, while the remaining address *E. coli*, *Shigella*, *Listeria*, and *Campylobacter*. These demonstrate significant potential requirements in combating *Salmonella* contamination in food safety and the urgent need for effective solutions to reduce foodborne illnesses associated with *Salmonella*. In contrast, there is a lack of approved phage products for human or animal use in EU [82]. Only Stafal is available in Slovakia and the Czech Republic against *Staphylococcus aureus* [83]. However, Stafal has not been evaluated under EU regulations because it entered the market before both countries joined the EU [82]. Overall, phage therapy offers several advantages, including its noninterference with mammalian cells/hosts, making it safe for humans and animals, and technological advancements lowering the costs of phage production compared to traditional antibiotic production [84, 85].

## 3. Limitation of Phage Application on *Salmonella*

### 3.1. Challenges in Phage Therapy for Host Cell

While the therapeutic applications of phages show immense promise, there are still several challenges, especially when the targeted host is *S. enterica*. The major obstacles include the emergence of phage resistance [84], gene transduction [86], and serotype or serovar variation of *S. enterica*. Phage cocktails, a combination of phages with different complementary properties, such as different host ranges, have been developed to combat the issue of variation in *S. enterica* [87]. Compared to single-phage treatments, which cause phage-resistant colonies, phage cocktails can eliminate bacteria while preventing the emergence of resistant strains [84]. However, phage cocktails may contain phages that target not only pathogenic bacteria but also beneficial bacteria in the microbiota. This could lead to unintended consequences, including microbiome disruption [88], and it can serve as a crucial mediator of genetic exchange between pathogenic and nonpathogenic bacteria [89, 90].

Lytic phages are generally preferred for therapeutic purposes. In contrast, lysogenic phages tend to be avoided due to their innate ability to promote gene transduction and potentially enhance bacterial pathogenicity by acquiring additional genes [84]. Although lytic phages have the potential to kill a significant portion of bacterial cells effectively, it is rare for them to completely eradicate the whole population of their host cells, implying that a small amount of the bacterial population employs multiple strategies to counteract invading phages [84]. These strategies include adsorption inhibition, restriction-modification (R/E) systems, utilization of plasmids, temperate genes, CRISPR–Cas (clustered regularly interspaced short palindromic repeats–CRISPR-associated proteins) systems [91], mobile genetic islands (capable of carrying genes encoding AMR) [61, 92], and abortive infection (Abi) [93]. These interventions enable bacteria to avoid phage invasion during various periods of the phage lifecycle.  Table 3 lists the obstacles related to using phage therapy to manage *S. enterica*, as well as suggested solutions and their drawbacks.

### 3.2. Immune Response of Animal/Human Organisms to Phages

Phages have been considered safe for humans and animals because they only infect prokaryotic cells rather than eukaryotic cells [48]. However, recent research has revealed that phages can elicit immune responses in eukaryotic cells and influence the effectiveness of phage therapy [94]. Repeated phage exposure can induce phage-specific neutralizing antibodies, which may accelerate the clearance of phages from tissues and reduce their effectiveness [95]. For instance, myophages, a phage type, induce a neutralizing antibody response compared to siphophages. This antibody response limits the ability to reuse the same phage treatment in an individual in the long term [95]. In addition to the humoral immune response, phages may interact directly with eukaryotic cells and induce cellular responses through receptors, such as TLR3 (for RNA phages) or TLR9 (for DNA phages) [96]. These interactions produce interferons and a process of innate immune response [97].

Phages can also shift microbiome patterns and increase gut permeability, causing endotoxemia and inflammation [98]. Phages may decrease the population of beneficial bacteria, such as *Lactobacillus* and *Faecalibacterium*, and increase potentially harmful species, such as *Butyrivibrio* and *Ruminococcus* [98]. Additionally, overdoses of phages could overburden the immune system of humans or animals, leading to possible unintended side effects [98]. Other limitations for phage therapy include restricted narrow specificity, stringent quality and safety standards, stability of preparations, effective assays in screening, activity limitations within biofilms, short lifespan within the animal body [99, 100], selection of suitable candidates, delivery to infection sites, and navigating regulatory approval [101]. Based on these challenges, scientists are exploring alternative approaches to address bacterial infections, such as phage-encoded endolysin (Table 3).

## 4. Approaches to Overcome the Limitation of Phage Therapy on Enteric Bacterial Pathogens

### 4.1. Endolysin as a Promising Alternative

Endolysins are muralytic enzymes produced by phages that hydrolyze bacterial cell walls, facilitating the release of progeny phages at the end of a lytic lifecycle [71]. In recent years, the potential of endolysins is due to their broad lytic effects on both Gram-positive and Gram-negative bacterial pathogens [102]. Endolysins are divided into five groups based on their distinct muralytic activities involving covalent bonds: (I) N-acetylmuramidase (lysozyme), (II) N-acetyl-*β*-d-glucosaminidase (glycosylase), (III) N-acetylmuramoyl-l-alanine amidase (amidases), (IV) l-alanoyl-d-glutamate endopeptidase (endopeptidase), and (V) interpeptide bridge-specific endopeptidase [103]. These enzymes target specific bonds within the peptidoglycan polymer of the bacterial cell wall, including glycosidic bonds (glycan portion of peptidoglycan), amide bonds (between the glycan moiety and the peptide moiety), and peptide bonds (within the stem peptide) [103]. While endolysins were initially used primarily on Gram-positive bacteria, recent studies have shown that utilizing endolysins as outer membrane permeabilizers (OMPs) has proven effective in eliminating Gram-negative bacteria [102].

Endolysins, autolysins, and exolysins are members of a small family of mammalian peptidoglycan recognition proteins [71, 104]. Autolysins primarily play a key role in remodeling peptidoglycan during cell growth and division, while exolysins are produced to eliminate competitive strains or species. Endolysin generally consists of two domains, including a cell wall-binding domain and an N-terminal enzymatic activity domain, enabling it to bind to a particular host cell wall and lyse the host cell wall [105]. The cell wall-binding domain identifies and specifically binds to the receptor of bacterial cell walls, and the enzymatic activity domain contributes to the cleavage of different peptidoglycan linkages [71]. The structure of phage endolysins differs between Gram-positive and Gram-negative specific phages. Most endolysins from Gram-positive specific phages consist of two domains, an enzymatic activity domain and a cell wall-binding domain, which are connected through a short flexible linker [106].

Unlike endolysins from Gram-positive specific phages, most endolysins from Gram-negative phages are small, globular, and single-domain proteins (range from 15 to 20 kDa) that usually lack a specific cell wall-binding domain [71]. Only a few endolysins from Gram-negative specific phages possess both a wall-binding domain and an enzymatic activity domain, which enable them to present a broad binding spectrum [71].

### 4.2. Effect of Endolysins Against *Salmonella* and Other Enteric Bacterial Pathogens

Endolysin has been demonstrated as a promising alternative against various AMR bacterial pathogens [102]. Since the first identification of endolysin from the *L. monocytogenes* phage in 1995, these enzymes have generated interest as a novel class of natural food preservatives [107]. Other studies have shown that endolysins are effective against many other Gram-positive bacteria, such as *Enterococcus faecalis* [108], *Streptococcus pneumoniae* [109], *Clostridium perfringens* [110], *S. aureus* [111], *Bacillus cereus* [112], and *L. monocytogenes* [113].

Endolysins perform specific, targeted bactericidal activity without disrupting beneficial or normal microbiota in ecosystems, including in the human and animal gut [114]. In addition, they have other benefits, such as quick lysis of bacterial cells, a low risk of resistance, effectiveness of synergistic function with other antibacterial agents, and the capacity to effectively act against biofilms and mucosal surfaces [114]. Due to these advantages, many purified or recombinant endolysins have been used to combat AMR bacteria, making them a promising alternative treatment [71].

Applications of exogenous endolysins against Gram-negative bacteria are limited due to the structure of their cell wall, which consists of a peptidoglycan layer, an internal cytoplasmic cell membrane, and an outer membrane with a lipopolysaccharide layer that prevents endolysins from accessing and lysing the peptidoglycan layer [115]. However, several recent reports have shown that endolysins can act against Gram-negative bacteria, including *S. enterica* [116–119], *Pseudomonas aeruginosa* [120], *Shewanella putrefaciens* [121], and *Vibrio parahaemolyticus* [122]. To facilitate the effectiveness against Gram-negative bacteria, these studies utilized OMPs, such as ethylenediaminetetraacetic acid (EDTA) [123], Triton X-100 [124], trichloromethane [124], organic acids [71], or the edible *ε*-poly-L-lysine (EPL) [125], to pretreat or cotreat the bacteria when applying exogenous endolysins. A recent study has shown that combining the fusion endolysin with KL-L9P, a sensitizer peptide known to enhance the effectiveness of antibiotics by remodeling the outer membrane of Gram-negative bacteria [126], could serve as a potential biocontrol agent against Gram-negative bacteria and *S. enterica* [127].

On the other hand, limited studies have revealed the effectiveness of endolysins using animal models [71]. Therefore, during the preclinical development of endolysins, it is imperative to address several key factors such as safety, toxicity, and immunogenicity. Despite numerous published animal trials, few endolysins have been tested in humans. Nevertheless, several studies have indicated no adverse health effects, elevated proinflammatory cytokine levels, or complement activation in mice, providing reassurance about the favorable safety and toxicity profiles of these endolysins [71]. So far, several clinical trials for phage-derived endolysins have reached various clinical trial phases, with some showing promising results (Table 4). However, all these trials have focused on the treatment of *S. aureus* rather than *Salmonella.*

Due to inherent limitations, there are not commercially available endolysin products targeting Gram-negative bacteria, including *Salmonella*.  Table 5 demonstrates research on phage-derived endolysin against *Salmonella* from 2014 to 2024. Despite the progress in developing these endolysins, they require a combination with OMPs or the use of fusion proteins to enhance their lytic ability and effectively penetrate the outer membrane of *Salmonella.*

## 5. Conclusion

The growing AMR bacteria in animals and humans highlights the urgent need to develop novel antimicrobial strategies. Phages and phage-encoded endolysin provide promising solutions with distinct antimicrobial mechanisms. Due to phage limitations, phage-encoded endolysins could be a more effective alternative. However, one main challenge for the use of endolysins against *S. enterica* is the outer membrane barrier for Gram-negative bacteria, which prevents the penetration of the endolysin and limits its efficacy of lytic activity. Therefore, despite *Salmonella* being the most common cause of foodborne disease globally, there is a lack of commercial endolysin products in the market or clinical applications. It emphasizes the need for continued innovation and development. Current and future efforts should focus on overcoming the outer membrane barrier, possibly through coadministration with permeabilizing agents or engineering endolysins with enhanced membrane penetration. Testing endolysin efficacy in animal models or food safety applications could pave the way for commercial use. Collaboration between academia and industry will be critical to advancing the development of endolysins for treating *Salmonella* and other resistant pathogens.

## Figures and Tables

**Table 1:** Benefits and limitations of different approaches against *Salmonella.*

	**Benefits**	**Limitations**
Probiotics	• Improves gut health • Enhances immunity • Reduces pathogen colonization • Synergistic effects • Competitive exclusion • Supports animal health • Minimal side effects	• Variable efficacy • Resistance to gastrointestinal conditions • Strain-specific effects • Gene transfer concerns • Potential toxicity • Shelf life and stability • Regulatory hurdles • Probiotic dosage issues

Prebiotics	• Enhances beneficial gut microbiota • Improves digestion • Supports synergistic action with probiotics • Reduces pathogen colonization • Improves growth performance in animals • Stabilizes gut pH • Nondigestible but nutritious • Safe for most populations	• Variable efficacy • Potential for increased pathogen susceptibility • No universal protection • Side effects • Limited research on long-term effects • Inconsistent synbiotic effects • Risk of pathogen translocation

Nature antimicrobial compounds	• Target-specific action • Broad and narrow spectrum • Natural and synthetic sources • Inhibition of virulence • Inhibition of biofilm formation • Minimal risk of resistance	• Development of resistance • Complexity of bacterial systems • Potential toxicity • Bioavailability issues • Cost and development time • Inconsistent efficacy • Short-term solutions

Essential oils	• Natural antimicrobial agents • Diverse applications • Broad range of therapeutic benefits • Synergistic effects with antibiotics • Plant-derived and ecofriendly	• Potential toxicity • Stability issues • Organoleptic effects • Volatility and solubility challenges • Regulatory and quality assurance issues

Vaccine	• Disease prevention • Long-lasting immunity • Safety • Innovative development	• Pathogen variability • Vaccine handling risks • Immunogenicity issues • Live attenuated vaccine risks • Complex development process

Phage (phage cocktails)	• Host specificity • Abundance in nature • Minor side effect for animals • Self-enrichment at the site of treatment • Stability and activity under various environmental conditions • Potential for genetic engineering	• Clearance by immune system • Resistance development of bacteria • Genetic instability • Regulatory approval • Deliver to the target site

Phage-derived endolysin	• No resistance development in bacteria • High efficacy in treating Gram-positive bacteria • Broader host range • Targets specific pathogenic bacteria without affecting microflora	• Short in vivo half-life • Reduced activity against Gram-negative bacteria (often requires a permeabilizer for Gram-negative treatment)

*Note: Source:* Lamichhane et al. [29].

**Table 2:** FDA-approved phage product for food pathogen control (as of 2024) [80].

**Product name**	**Company**	**GRN no.**	**Target pathogens**	**Approval year**	**Applications**
*Salmonella* Enteritidis Phage Preparation (Strain SP8)	Qingdao Phagepharm Bio-Tech Co. Ltd.	1134	*Salmonella* Enteritidis	2024	Ground chicken
Applied Phage Vegetable S2	FINK TEC GmbH, Germany	1070	*Salmonella enterica*	2023	Fresh and processed fruit and vegetables
Applied Phage Meat S2	FINK TEC GmbH, Germany	1038	*Salmonella enterica*	2023	Ground and whole red meat and poultry, including whole carcasses, primals, subprimals, trimmings, and organs
CampyShield	Intralytix Inc., United States	966	*Campylobacter jejuni*	2021	Raw red meat, including whole carcasses, primals, subprimals, cuts, trimmings, and organs, and raw poultry, including carcasses and parts
GPI Biotech VAM-S	Gum Products International, Canada	917	*Salmonella enterica*	2020	Poultry, eggs, red meat, fruits, vegetables, fish, and shellfish
EcoShield	Intralytix Inc., United States	834	*Escherichia coli*	2019	Ground and whole meat and poultry, including whole carcasses, primals and subprimals, trimmings, and organs; RTE meats and poultry; fresh and processed fruits; fresh and processed vegetables; dairy products (including cheese); and fish and other seafood
ECLYPSE-STEC	OmniLytics, Inc., United States	827	*Escherichia coli*	2019	Poultry, red meats, fruits, vegetables, eggs, fish, and shellfish
Phage Guard E	Micreos Food Safety Inc., the Netherlands	757	*Escherichia coli* O157	2018	Beef carcasses, subprimals, beef cuts, and trimmings intended for ground beef
SalmoPro	Phagelux Inc., Canada	752	*Salmonella enterica*	2018	Poultry, red meats, fruits, vegetables, eggs, fish, and shellfish
FinaLyse	FINK TEC GmbH, Germany	724	*Escherichia coli*	2018	Beef carcasses
ShigaShield (ShiActive)	Intralytix Inc., United States	672	*Shigella* spp.	2017	Dairy products; RTE meats; fresh and processed vegetables; and fresh and processed fruits, fish, and shellfish
SalmoPro	Phagelux Inc., Canada	603	*Salmonella enterica*	2016	As an antimicrobial to control *Salmonella* in food
ListShield	Intralytix Inc., United States	528	*Listeria monocytogenes*	2014	Dairy products, fresh and processed vegetables, fresh and processed fruits, and fish and shellfish
Salmonelex	Micreos B.V., the Netherlands	468	*Salmonella enterica*	2013	Pork and poultry products, beef, vegetables, and fresh and saltwater seafood (excluding *Siluriformes* [catfish])
SalmoFresh	Intralytix Inc., United States	435	*Salmonella enterica*	2013	RTE and raw poultry products; RTE and raw red meat carcasses, subprimals, and trimmings; fish; shellfish; and fresh and processed fruits and vegetables
Listex P100	EBI Food Safety B.V., the Netherlands	218	*Listeria innocua*	2007	Meat and poultry products
Listex P100	EBI Food Safety., the Netherlands	198	*Listeria innocua*	2006	Cheeses are normally aged and ripened

Abbreviation: RTE, ready-to-eat.

**Table 3:** Challenges in phage therapy for controlling *Salmonella enterica.*

**Challenge**	**Potential solutions**	**Risks/limitations**
Phage resistance	Phage cocktails with multiple complementary phages	Possible emergence of new resistant strains; difficulty in completely eradicating bacteria
Gene transduction	Prefer the use of lytic phages that do not engage in gene transduction	Some lytic phages may not fully eliminate bacteria; phage-resistant populations may remain
Serotype/serovar variation	Use of phage cocktails targeting different serotypes	Possible disruption of beneficial microbiota; unintended effects on nonpathogenic bacteria
Impact on microbiota	Develop highly specific phages or tailored cocktails	Microbiome disruption may lead to unintended health effects or imbalanced gut flora
Defense mechanisms (adsorption inhibition, CRISPR-Cas, etc.)	Combination of phages with alternative therapies, for example, antibiotics	Bacterial defense mechanisms may reduce overall treatment efficacy, requiring ongoing monitoring

**Table 4:** Clinical trials for phage-derived endolysin [128, 129].

**Products**	**Target bacteria**	**Sponsor**	**ClinicalTrials.gov ID**	**Clinic trial phase**	**Trial start**	**Last verified**	**Clinical outcome**	**Result publication**
HY-133	*S. aureus*	University Hospital Tuebingen	NCT06290557	Phase 1	2024-07	2024-03	Ongoing	Not available
Tonabacase (LSVT-1701)	*S. aureus*	Lysovant	NCT05329168	Phase 2	2022-05	2022-06	No serious adverse events (AEs) reported	Not available
CF-301	*S. aureus*	ContraFect	NCT03163446	Phase 2	2017-05	2021-09	No serious AEs reported	[130]
N-Rephasin SAL200	*S. aureus*	Intron Biotechnology Inc.	NCT03089697	Phase 2	2017-02	2021-10	No serious AEs reported	[131]
Staphefekt SA.100	*S. aureus*	Erasmus Medical Center	NCT02840955	Not applicable	2016-06	2018-02	No serious AEs reported	[132]
CF-301	*S. aureus*	ContraFect	NCT02439359	Phase 1	2015-05	2020-04	No serious AEs reported	Not available
N-Rephasin SAL200	*S. aureus*	Intron Biotechnology Inc.	NCT01855048/NCT03446053	Phase 1	2013-05	2021-11	No serious AEs reported	[133]
P128	*S. aureus*	GangaGen Inc.	NCT01746654	Phases 1 and 2	2012-12	2016-03	No serious AEs reported	Not available

*Note: Source:* National Library of Medicine (https://clinicaltrials.gov). Phase 1: Clinical trials involve testing a new treatment to assess its safety, potential side effects, optimal dosage, and timing. These trials typically involve 20–100 healthy volunteers or individuals with the disease/condition, lasting several months. Phase 2: Clinical trials evaluate the efficacy and side effects of an experimental drug for a specific disease or condition in approximately 100–300 participants.

Abbreviation: AEs, adverse events.

**Table 5:** Phage-derived endolysin tested to inhibit growth of *Salmonella* (from 2014 to 2024).

**Target *Salmonella* strains**	**Phage endolysin**	**Cotreatment**	**Published date**	**Endolysin source**	**Clone/vector system**	**Induction**	**Effectiveness**
*S.* Enteritidis	ENDO-1252 [134]	0.1 mM EDTA	2024	*Salmonella* bacteriophage-1252	BL21 (DE3)/pET28c	0.1 mM IPTG at 25°C for 18 h	A 6-h treatment with 120 *μ*g of ENDO-1252, combined with 0.1 mM EDTA at 25°C and pH 7.0, resulted in a 1.15 log reduction (92.87%) of *S.* Enteritidis
*S.* Typhimurium	LysKpV475 [135]	Polymyxin B and *Salmonella* bacteriophage phSE-5	2024	*Klebsiella* phage vB_KpnP_KpV475	BL21 (DE3)/pET29b	0.1 mM IPTG at 16°C for 16 h	LysKpV475 showed an enhanced bacteriostatic effect when combined with polymyxin B. A synergistic antimicrobial effect was also observed when paired with the phage phSE-5, further boosting its activity. Moreover, immobilizing LysKpV475 in a pullulan matrix led to a 2-log reduction of *Salmonella* after just 6 h of treatment
*S.* Enteritidis and *S.* Typhimurium	Lys1S-L9P [127]	Fuse with sensitizer peptide L9P	2023	Bacteriophage SPN1S	BL21 (DE3)/pET28a	0.5 mM IPTG at 18°C for 18 h	Treatment with 1 *μ*M of the fused endolysin, Lys1S-L9P, at 37°C for 1 h reduced 5 logs for *S.* Enteritidis and 4 logs for *S.* Typhimurium
*S.* Blukwa (NCTC 8271)	rLysJNwz [136]	0.5 mM EDTA	2023	Bacteriophage JNwz02	BL21/pColdI	0.2 mM IPTG at 15°C for 24 h	rLysJNwz in combination with 0.5 mM EDTA was assessed on contaminated eggs and lettuce for 1 h. This treatment reduced viable *Salmonella* by more than 86.7% on eggs and 86.5% on lettuce
*S*. Typhimurium	ST01 [137]	Fuse with cecropin A gene (CecA::ST01)	2022	*Salmonella* Typhimurium phage PBST08	BL21 (DE3)/pET21a	1 mM IPTG at 37°C for 5 h for ST01; 1 mM IPTG at 18°C for 21 h for CecA::ST01	Reduce *S*. Typhimurium by 4 logs with 1 *μ*M of CecA::ST01
*S*. Pullorum	LySP2 [138]	*Pichia pastoris* (*Komagataella phaffii*)	2022	*Salmonella* phage YSP2	*Pichia pastoris* X33/pPICZ-*α*A	Buffered minimal methanol medium at 29°C for 72–96 h. One percent (*v*/*v*) methanol was added to the medium every 12 h	Treatment with LySP2 can enhance the survival rate of *Salmonella*-infected chicks to as high as 70% while also decreasing the presence of *Salmonella* in both the liver and intestine
*S.* spp.	Salmcide-p1 [139]	Pretreat with 100 mM EDTA	2022	*Salmonella* phage fmb-p1	BL21 (DE3)/pT7-GST-His	1 mM IPTG at 16°C for 16 h	Salmcide-p1 exhibited broad bactericidal activity against Gram-negative bacteria, demonstrating a wider anti-*Salmonella* spectrum than phage fmb-p1. When combined with EDTA-2Na, Salmcide-p1 effectively suppressed the growth of Gram-negative bacteria inoculated in skim milk
*S*. spp.	XFII [140]	Chitosan	2022	*Salmonella* phage XFII-1	BL21 (DE3)/pET-29b	1 mM IPTG at 16°C for 4 h	The ideal synergistic effect was achieved with 60 *μ*g/mL of XFII in combination with 0.375 mg/mL of chitosan, resulting in a decrease in OD_600nm_ from 0.88 to 0.58 within 10 min
*S*. Typhimurium	LysSTG2 [141]	Slightly acidic hypochlorous water (SAHW) (40 mg/L chlorine)	2021	*Salmonella* phage STG2	BL21 (DE3)/pET29b	0.25 mM IPTG at 16°C for 16 h	After a 1 h treatment with LysSTG2 (100 *μ*g/mL), the viability of *S*. Typhimurium was significantly reduced, showing a reduction of 1.2 logs. Furthermore, when SAHW with 40 mg/L available chlorine was combined with LysSTG2 (100 *μ*g/mL), it effectively eliminated more than 99% of *S.* Typhimurium biofilm cells
*S*. Enteritidis, *S*. Typhimurium, Argora, Indiana, Anatum, and Dublin	LysSE24 [142]	Pretreat with 100 mM EDTA	2020	*Salmonella* phage LPSE1	BL21 (DE3)/pET28b; *E. coli* C41/pET29b	1 mM IPTG at 16°C for 16 h, 30°C for 4 h, and 37°C for 4 h, respectively	Activity against all tested Gram-negative bacterial strains in the presence of OMPs; potential role in the food industry
*S*. Typhimurium	LysWL59 [123]	0.5 mM EDTA	2019	Bacteriophage LPST10	BL21 (DE3)/pET28b; *E. coli* C41/pET29b	0.25 mM IPTG at 16°C for 12 h, 30°C for 4 h, and 37°C for 4 h, respectively	Reduction of *S*. Typhimurium on lettuce, especially in the presence of 0.5 mM EDTA (postharvest)
*S.* Enteritidis (ATCC13076)	LyS15S6 [125]	e-poly-L-lysine (EPL)	2019	*Salmonella*-virus-FelixO1 phage BPS15S6	BL21 (DE3)/pET28a	1 mM IPTG at 37°C for 4 h	When combined with 1 *μ*g/mL EPL, 2 *μ*M LyS15S6 resulted in 2.56 and 3.14 log reductions of *S.* Enteritidis after 15 min of reaction at 25°C and 2 h of reaction at 8°C, respectively
*S.* Typhimurium	BSP16Lys [143]	Liposome	2019	Phage BSP16	BL21 (DE3)/pET28a	0.5 mM IPTG at 37°C for 3 h	When S. Typhimurium cells were treated with BSP16Lys-encapsulated liposomes, they exhibited 2.2 and 1.6 log reductions, respectively, without a membrane permeabilizer
*S.* Typhimurium	ABgp46 [144]	Citric and malic acid	2016	Acinetobacter phage vb_AbaP_CEB1	BL21 (DE3)/pET15b	0.5 mM IPTG at 16°C for overnight	ABgp46 reduces more than 4 logs of *S.* Typhimurium when citric and malic acid are present
*S.* Typhimurium	Lys68 [145]	Malic or citric acid	2014	*Salmonella* phage phi68	BL21 (DE3)/pET28a	0.5 mM IPTG at 16°C for 18 h	Maximal reduction of 5 logs *S*. Typhimurium after 2 h treatment with Lys68/citric acid

*Note:* BL21, competent *E. coli* cell.

Abbreviation: IPTG, isopropyl-*β*-D-thiogalactopyranoside.

## Data Availability

The data that support the findings of this study are available from the corresponding author upon reasonable request.
